# Effect of MAP3K8 on Prognosis and Tumor-Related Inflammation in Renal Clear Cell Carcinoma

**DOI:** 10.3389/fgene.2021.674613

**Published:** 2021-09-10

**Authors:** Jiatao Hao, Yumeng Cao, Hui Yu, Lu Zong, Ruifang An, Yan Xue

**Affiliations:** ^1^Department of Obstetrics and Gynecology, The First Affiliated Hospital of Xi’an Jiaotong University, Xi’an, China; ^2^Graduate School of China Medical University, China Medical University, Shenyang, China

**Keywords:** MAPK kinase kinase 8, expression, methylation, prognosis, protein-protein interaction, functional enrichment analysis

## Abstract

**Background:** MAPK kinase kinase 8 (MAP3K8) is involved in the regulation of MAPK cascades and immune responses. Differential expression of MAP3K8 is closely correlated with tumorigenesis. In this study, we used bioinformatics tools to explore expression level, prognostic values, and interactive networks of MAP3K8 in renal clear cell carcinoma (ccRCC).

**Methods:** Differential expression of MAP3K8 was determined by TIMER2.0, UALCAN, and Oncomine Platform. For exploration of MAP3K8 mutation profile, TIMER2.0, DriverDBv3, and cBioPortal were used. The survival module of GEPIA, UALCAN, and DriverDBv3 was used to examine the prognostic value of MAP3K8. Immune infiltration was estimated by TIMER, TIDE, CIBERSORT, CIBERSORT-ABS, QUANTISEQ, XCELL, MCPCOUNTER, and EPIC algorithms. PPI networks and functional enrichment analysis were constructed using GeneMANIA, Cytoscape, and Metascape. The co-expression module in cBioPortal was used to find genes that are correlated with MAP3K8 in mRNA expression.

**Results:** Compared to normal renal samples, ccRCC (3.08-fold change, *P* = 1.50E-7; 1.10-fold change, *P* = 3.00E-3), papillary RCC (2.24-fold change, *P* = 1.86E-4), and hereditary ccRCC (1.98-fold change, *P* = 1.69E-9) have significantly higher levels of MAP3K8 expression. Compared to Grade 1 ccRCC samples, Grade 2 (*P* = 1.28E-3) and Grade 3 (*P* = 7.41E-4) cases have higher levels of MAP3K8 methylation. Percentage of patients harboring MAP3K8 mutation is 0.3% from TIMER2.0 and 0.2 to 11.5% from cBioPortal. High levels of MAP3K8 expression were associated with poorer overall survival (OS) in ccRCC (GEPIA: Log-rank *P* = 0.60E-2, HR = 1.5; DriverDBv3: Log-rank *P* = 1.68E-7, HR = 2.21; UALCAN: *P* = 0.20E-2). MAP3K8 was positively correlated with the presence of T cell regulatory (Tregs) (QUANTISEQ: Rho = 0.33, *P* = 1.59E-13). PPI network and functional enrichment analyses revealed that MAP3K8 correlated with NFKBIZ, MIAT, PARP15, CHFR, MKNK1, and ERMN, which was mainly involved in I-kappaB kinase/NF-kappaB and toll-like receptor signaling pathways.

**Conclusion:** MAP3K8 overexpression was correlated with damaged survival in ccRC and may play a crucial role in cancer-related inflammation via I-kappaB kinase/NF-kappaB and toll-like receptor signaling pathways.

## Introduction

Renal cell carcinoma (RCC) encompasses a heterogeneous group of malignancies originated from renal tubular epithelial cells and represents the tenth most commonly diagnosed cancer, which approximately claimed 270,000 new cases and 116,000 deaths annually worldwide (Ljungberg et al., 2019; Siegel et al., 2019). Clear cell RCC (ccRCC) accounts for 80% of all RCC diagnoses and is the most common cause for RCC-associated death and therefore is the focus of this study (Escudier et al., 2019). Localized RCC can be treated with surgery mainly including partial and radical nephrectomy with curative intent (Ljungberg et al., 2019). However, around one third of patients with localized RCC inevitably develop metastases, which are those with the worst prognosis and require systemic therapy (Escudier et al., 2019). Although target therapies against vascular endothelial growth factor (VEGF) (Yang et al., 2003; Escudier et al., 2010) and mammalian target of rapamycin (mTOR) (Escudier et al., 2007; Motzer et al., 2007) pathways have been approved for the treatment of metastatic RCC, as well as immunotherapy using immune checkpoint blockade with monoclonal antibodies (Motzer et al., 2015, 2018), most patients with advanced disease will eventually progress with shorter survival time. In the last decades, backed up by a continuing development of genomic and molecular profile of metastatic ccRCC, an unprecedented number of new drugs with different effective mechanisms of action has been tested in clinical trials. In addition, genetic researches are also beginning to be conducted to identify biomarkers related to clinical outcomes, and unveil complicated biological processes and molecular mechanism that lead to unfavorable prognosis of ccRCC patients (Hsieh et al., 2017). Large-scale genomic projects have suggested that prevalence of germline and cancer-predisposing mutations were higher among patients with ccRCC compared with normal tissues, including VHL, PBRM1, SETD2, and BAP1, whereas how these mutations contribute to the pathogenesis and values as prognostic biomarkers in ccRCC are largely unknown (Peña-Llopis et al., 2012; Cancer Genome Atlas Research Network, 2013; Sato et al., 2013; Xu et al., 2016). Hence exploring more potential therapeutic targets and predictive biomarkers is of urgent need for ccRCC.

Mitogen-activated protein kinase (MAPK) cascade leading to activation of mitogen-activated extracellular signal-regulated kinase (MEK), extracellular signal-regulated kinases (ERK), and c-Jun N-terminal kinases (JNK), is involved in regulating the tumorigenesis and inflammatory diseases (Yuan et al., 2020). Therefore, MAPK pathway is long been perceived as attractive for new cancer therapy targets. As a main upstream in MAPK signaling transduction, MAPK kinase kinase 8 (MAP3K8), which is also known as tumor progression locus 2 (Tpl2), can activate a plethora of downstream molecules including MEK, ERK, and JNK to influence the growth and survival of cancer cells in concert with other signaling pathways, such as nuclear factor kappa-B (NFKB), tumor necrosis factor (TNF), and interleukin-1 (IL-1) (Lee et al., 2015a; Braicu et al., 2019). Previous studies demonstrated that MAP3K8 could induce progression of prostate and hepatocellular cancer through activation of C-X-C chemokine ligand (CXCL), FAK/AKT signaling pathway, and interleukins (Lee et al., 2015b; Li et al., 2015). However, whether MAP3K8 has deleterious or beneficial effects on tumor progression and clinical outcome of cancer patient is still highly controversial because of its multifaced roles and cross-talks with other molecular linchpins. Dysregulation of MAP3K8 has been observed in different tumors (Tunca et al., 2013; Boldrini et al., 2017; Lehmann et al., 2019), but there is little evidence about the role of MAP3K8 in ccRCC. Herein, we conducted a computational method for dissecting the relationships among genomic alterations of MAP3K8 and patient outcome, as well as protein-protein interaction (PPI) between MAP3K8 and other molecules in ccRCC patients. To the best of our knowledge, this is the first study to comprehensively identify the aberrantly expressed MAP3K8 of prognostic significance and PPI based on large-scale data and data mining of ccRCC.

## Materials and Methods

### Identifying Differential Expression of MAP3K8

To determine the differential expression of MAP3K8 across diverse cancer types, TIMER2.0 (Li et al., 2020)^1^ and UALCAN (Chandrashekar et al., 2017)^2^ were used, which were user-friendly and interactive web resources for analyzing genomic features among cancers. Users just need to enter a gene name in the analysis page to perform pan-cancer gene expression analysis. Distributions of MAP3K8 expression level are displayed using box plots in TIMER2.0 and UALCAN. Users can identify genes that are up-regulated or down-regulated in the tumors compared to normal tissues for each cancer type. The statistical significance computed by the Wilcoxon test is annotated by the number of stars (^∗^*P*-value < 0.05; ^∗∗^*P*-value < 0.01; ^∗∗∗^*P*-value < 0.001). In addition, UALCAN was also applied to explore expression of MAP3K8 based on clinicopathologic factors including sample types, cancer stage, age, gender, tumor grade, and metastasis status. Results were displayed using Box-Whisker plots. Significance of difference is estimated by Student’s *t*-test, and *P*-value < 0.05 is considered significant. For generation of MAP3K8 mRNA expression level between ccRCC and normal kidney samples, Oncomine Platform (Rhodes et al., 2004),^3^ which includes more than 700 high-quality datasets consisting of approximately 87,000 cancers and normal samples, was used. Visualization of statistics of MAP3K8 available in Oncomine Platform is presented with bar graph using Student’s *t*-test with *P*-value < 0.01 and fold change >1.

### Exploring Gene Mutation Profile of MAP3K8

For exploration of MAP3K8 gene mutation profile, TIMER2.0, DriverDBv3 (Liu et al., 2020),^4^ and cBioPortal (Gao et al., 2013)^5^ were used, which provide a web resource for exploring, visualizing, and analyzing multidimensional cancer genomics data. The Gene-mutation module of TIMER2.0 and DriverDBv3 was first applied to conduct a comprehensive analysis of MAP3K8 mutation across TCGA cancers. Then, modules of cancer types summary and mutations in cBioPortal were screened for somatic mutation frequency and mutation types of MAP3K8 in ccRCC.

### Examining Prognostic Potential of MAP3K8

The survival module of GEPIA (Tang et al., 2017),^6^ UALCAN, and DriverDBv3 was used to examine the prognostic potential of MAP3K8 based on expression level in ccRCC. GEPIA is a newly developed interactive web server for analyzing the RNA sequencing expression data of 9,736 tumors and 8,587 normal samples from TCGA. UALCAN could provide customizable survival analysis based on clinicopathologic factors, such as tumor grade, patient race, and gender. Survival endpoints including overall survival (OS), disease-free interval (DFI), progression-free interval (PFI), and disease-specific survival (DSS) were provided in the datasets; however, we only chose OS as the endpoint in this study. The ccRCC samples were stratified into 2 groups: samples with highly expressed MAP3K8 (red) and samples with lowly expressed MAP3K8 (blue or green). Log-Rank *P*-value, which was considered significant if it was less than 0.05, and hazard ratio (HR) were provided in the Kaplan-Meier plots.

### Visualizing the Correlation of MAP3K8 Expression With Immune Infiltration

TIMER2.0 could provide immune infiltration estimations for users-provided expression profiles by multiple immune deconvolution methods including TIMER, TIDE, CIBERSORT, CIBERSORT-ABS, QUANTISEQ, XCELL, MCPCOUNTER, and EPIC algorithms, and allow users to generate high-quality figures dynamically to explore tumor immunological features comprehensively (Li et al., 2020). Gene module of TIMER2.0 allows users to select any gene of interest and visualize the correlation of its expression with immune infiltration level in diverse cancer types. Once MAP3K8 gene and subtype of immune infiltrates submitted, a heatmap with numbers will show the purity-adjusted spearman’s rho across various cancer types. When you click your interested cell on the heatmap, a scatter plot will pop out to present the relationship between infiltrates estimation value and MAP3K8 expression. Furthermore, positive and negative correlation of MAP3K8 expression with immune infiltrates in ccRCC with significant statistics were selected. In addition, tumor purity is a major confounding factor in this analysis, since most immune cell types are negatively correlated with tumor purity. Therefore, we selected the “Purity Adjustment” option, which will use the partial Spearman’s correlation to perform this association analysis. Specially, for methods like EPIC and quanTIseq, which provide cell fractions referred to total cells, tumor purity and immune infiltration are necessarily negatively correlated, hence there is no need to adjust purity for the association analysis using the estimations from EPIC and quanTIseq.

### Constructing PPI Networks and Functional Enrichment Analyses

GeneMANIA (Warde-Farley et al., 2010)^7^ was first used to find gene lists related to MAP3K8 based on a variety of sources. This user-friendly online tool can construct networks of the genes related to MAP3K8 by the bioinformatics methods, including protein and genetic interactions, pathways, co-expression, co-localization, and protein domain similarity. In addition, functional enrichment analysis of MAP3K8-related genes was also displayed. Cytoscape (Shannon et al., 2003)^8^ was also used to visualize the results of PPI networks by searching MAP3K8 gene in PSICQUIC service and importing PPI records in BioGrid database. In Metascape (Zhou et al., 2019),^9^ a Cytoscape MCODE plugin-in was used to provide access to select hub modules of PPI network of MAP3K8. The interactive gene list of MAP3K8 constructed by GeneMANIA was input into Metascape for further function and enrichment pathways. We used Metascape to perform Gene Ontology (GO) analysis including biological process (BP), cellular component (CC), and molecular function (MF), and Kyoto Encyclopedia of Genes and Genomes (KEGG) pathway analysis of MAP3K8. *P*-value < 0.05 was considered as statistically significant.

### Finding Correlated Genes of MAP3K8

The co-expression module in cBioPortal was used to find genes that are correlated with MAP3K8 in mRNA expression. Co-expression and correlation of the top 25 genes associated with MAP3K8 gene in ccRCC were selected. Gene name and cytoband of correlated genes were showed with statistics of Spearman’s correlation. *P*-value is derived from two-sided *t*-test and the *q*-value derived from Benjamini-Hochberg FDR correction procedure.

## Results

### MAP3K8 Expression Profile

To find the overall expression profile of MAP3K8 across all TCGA cancers, firstly, we studied the transcriptome difference of MAP3K8 between tumor and normal samples using TIMER2.0. According to *P*-value computed by the Wilcoxon test, results showed that MAP3K8 was upregulated in cholangiocarcinoma (CHOL), colon adenocarcinoma (COAD), glioblastoma multiforme (GBM), liver hepatocellular carcinoma (LIHC), kidney renal clear cell carcinoma (KIRC, ccRCC), and stomach adenocarcinoma (STAD) and downregulated in bladder urothelial carcinoma (BLCA), breast invasive carcinoma (BRCA), head and neck squamous cell carcinoma (HNSC), kidney chromophobe (KICH), lung adenocarcinoma (LUAD), lung squamous cell carcinoma (LUSC), pancreatic adenocarcinoma (PAAD), pheochromocytoma and paraganglioma (PCPG), prostate adenocarcinoma (PRAD), thyroid carcinoma (THCA), and uterine corpus endometrial carcinoma (UCEC) (Supplementary Figure 1 and Supplementary Table 1).

**FIGURE 1:**
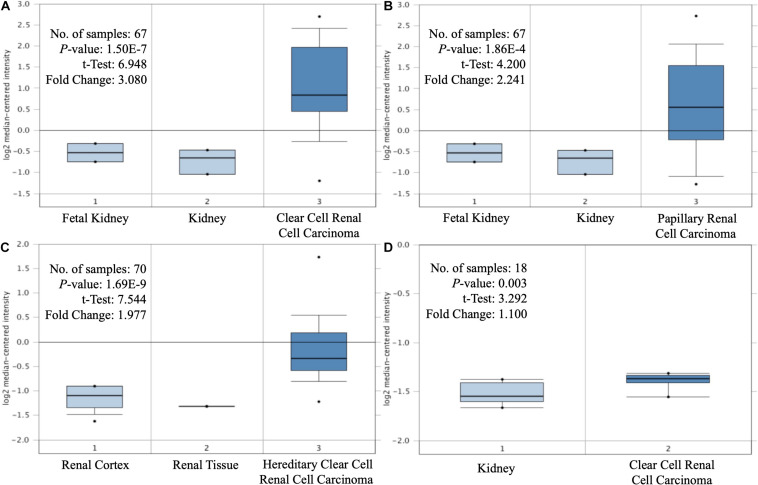
Differential expression of MAP3K8. Box-plot comparison of MAP3K8 expression for Yusenko clear cell renal cell carcinoma dataset **(A)**, Yusenko papillary renal cell carcinoma dataset **(B)**, Beroukhim hereditary clear cell renal cell carcinoma dataset **(C)**, and Lenburg clear cell renal cell carcinoma dataset **(D)**.

**FIGURE 2:**
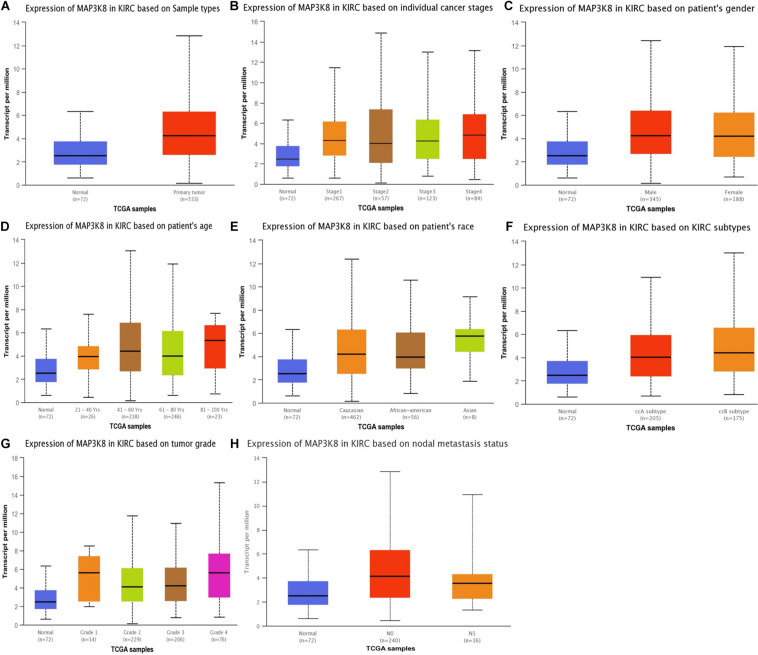
Expression of MAP3K8 in ccRCC for different clinicopathological parameters. Box plots showing relative expression of MAP3K8 in panel for sample types **(A)**, individual cancer stages **(B)**, patient’s gender **(C)**, patient’s age **(D)**, patient’s race **(E)**, ccRCC subtypes **(F)**, tumor grade **(G)**, and nodal metastasis status **(H)**.

The expression levels of MAP3K8 in normal kidney tissue, ccRCC, and papillary RCC samples were compared. Compared to normal renal samples, ccRCC (3.08-fold change, *P* = 1.50E-7; 1.10-fold change, *P* = 3.00E-3), papillary RCC (2.24-fold change, *P* = 1.86E-4), and hereditary ccRCC (1.98-fold change, *P* = 1.69E-9) have significantly higher levels of MAP3K8 expression (Figure 1). Given the dominating role, we focused further investigations on ccRCC and, in particular, on its associations with MAP3K8. To explore expression profile of MAP3K8 based on clinicopathological parameters in ccRCC, subgroup analyses were performed using UALCAN (Figure 2 and Supplementary Table 2). In addition to that ccRCC patients with different clinical features had higher levels of MAP3K8 expression than the normal, we found that patients with ccB (*P* = 8.77E-4) and tumor grade 4 (*P* = 2.42E-2) subtypes expressed more MAP3K8 than that with ccA and tumor grade 3, respectively. These findings indicate that MAP3K8 expression may be correlated with aggressive ccRCC tumors.

### Promoter Methylation Level of MAP3K8

**FIGURE 3:**
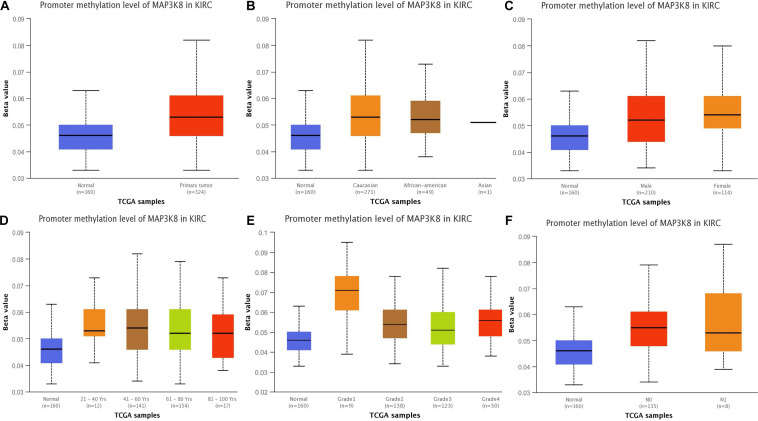
Promoter methylation of MAP3K8 in ccRCC for different clinicopathological parameters. Box-plot showing relative promoter methylation level of MAP3K8 in panel for sample types **(A)**, patient’s race **(B)**, patient’s gender **(C)**, patient’s age **(D)**, tumor grade **(E)**, and nodal metastasis status **(F)**.

To better understand the important roles of MAP3K8 methylation in oncogenesis and progression, we explored methylation levels of MAP3K8 in different ccRCC samples, including tumor status (normal and tumor), patient race (Caucasian, African-American, and Asian), gender (male and male), patient age (21–40, 41–60, 61–80, and 81–100 years), tumor grade (Grade 1, Grade 2, Grade 3, and Grade 4), and metastasis status (N0 and N1). The results are shown as box plots in Figure 3, suggesting that MAP3K8 methylation are significantly related to tumor status and tumor grade (Supplementary Table 3). MAP3K8 methylation were significantly abnormally expressed in ccRCC samples (*P <* 1E-12). Compared to Grade 1 ccRCC samples, Grade 2 (*P* = 1.28E-3) and Grade 3 (*P* = 7.41E-4) ccRCC samples have higher levels of MAP3K8 methylation, while race, gender, age, and nodal metastasis were not implicated in influencing MAP3K8 methylation.

### MAP3K8 Mutation Profile

**FIGURE 4:**
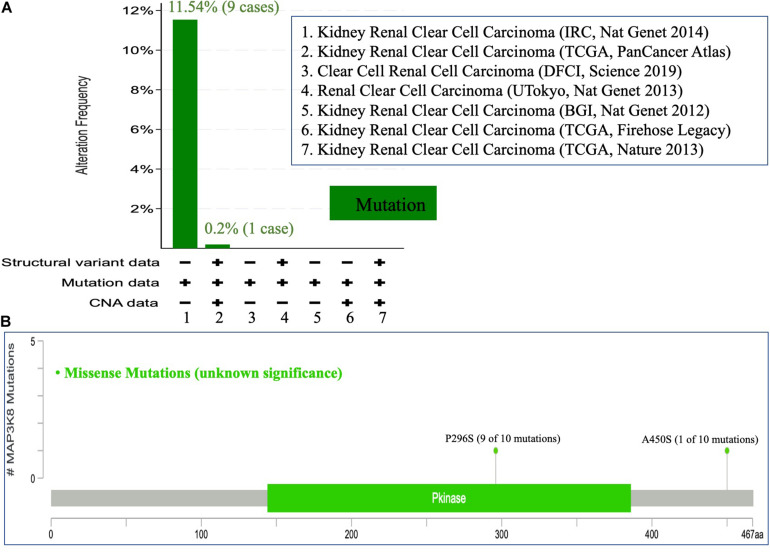
Mutational analysis of MAP3K8. Mutation of MAP3K8 along cancer studies **(A)**. Genomic information of MAP3K8 mutation **(B)**.

To comprehensively investigate the mutation profile of MAP3K8 across all TCGA cancers, TIMER2.0 and DriverDBv3 were used, suggesting that MAP3K8 mutation is rare among most cancers with frequencies ranging from 0.1% (1 of 500 THCA patients) to 4.5% (24 of 531 UCEC patients) (Supplementary Figure 2). We observed that the percentage of ccRCC patients harboring MAP3K8 mutation is 0.3% (1 of 370 patients) from the TIMER2.0 dataset (Supplementary Figure 2A), whereas data from cBioPortal server suggested that 0.2% (1 of 511) to 11.5% (9 of 78) ccRCC patients had somatic MAP3K8 mutation (Figure 4A). Markedly, 9 of 10 were duplicated mutations with unknown significance, resulting in changes of P296S in protein kinase domain (144–386) (Figure 4B).

### Prognostic Potential of MAP3K8

**FIGURE 5:**
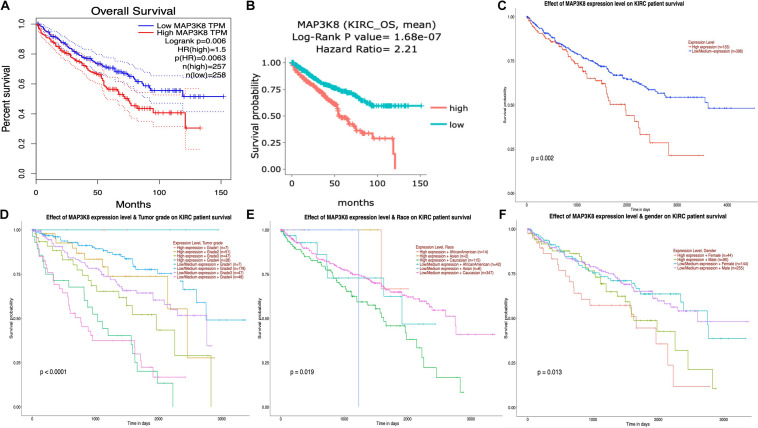
Kaplan-Meier plot for survival assay of MAP3K8 in ccRCC. Effect of MAP3K8 expression on overall survival from GEPIA **(A)**, DriverDBv3 **(B)**, and UALCAN **(C)** database. Effect of MAP3K8 expression on overall survival based on tumor grade **(D)**, patient’s race **(E)**, and patient’s gender **(F)** from UALCAN database.

The interrelationships between MAP3K8 expression and OS in ccRCC were retrieved from GEPIA, DriverDBv3, and UALCAN databases to construct Kaplan-Meier plots. Results revealed that high levels of MAP3K8 expression were associated with poorer prognosis of OS in ccRCC (GEPIA: Log-rank *P* = 0.60E-2, HR = 1.5; DriverDBv3: Log-rank *P* = 1.68E-7, HR = 2.21; UALCAN: *P* = 0.20E-2) (Figures 5A–C). Few significant interactions were observed between OS and clinicopathological features, indicating that tumor grade (*P <* 1.0E-4), patient race (*P* = 1.9E-2), and patient gender (*P* = 1.3E-2) may influence the prognosis of ccRCC patients with high-level MAP3K8 expression (Figures 5D–F).

### Coexpression and Correlation of Genes and Pathways Associated With MAP3K8

**FIGURE 6:**
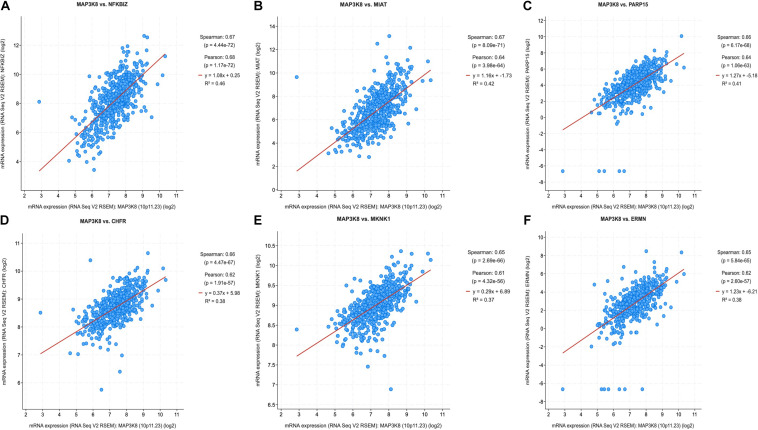
Coexpression of genes correlated with MAP3K8 in ccRCC based on cBioPortal. Graphical representation of Spearman’s correlation test of MAP3K8 gene with NFKBIZ **(A)**, MIAT **(B)**, PARP15 **(C)**, CHFR **(D)**, MKNK1 **(E)**, ERMN **(F)**.

**FIGURE 7:**
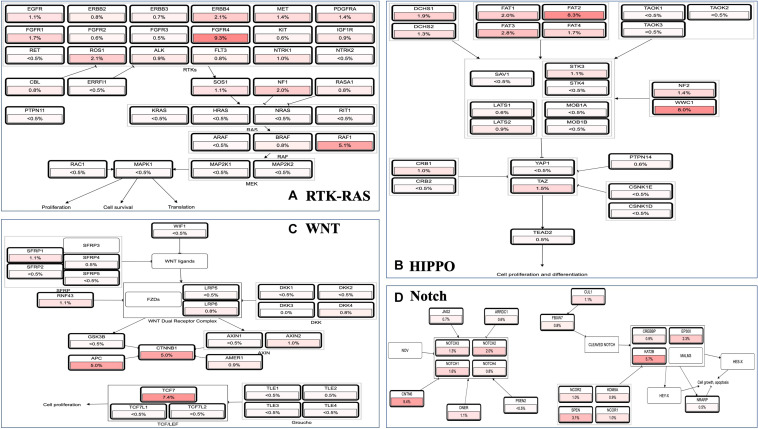
Pathway analyses in ccRCC using cBioPortal server. Impact of MAP3K8 and associated genes in regulating alteration frequency of RTK-RAS signaling pathway **(A)**, HIPPO signaling pathway **(B)**, WNT signaling pathway **(C)**, and Notch signaling pathway **(D)**.

Because MAPK cascades have shown multifaced roles and cross-talks with other molecular linchpins, we aimed to determine the correlation of MAP3K8 expression with the expression of other genes in ccRCC. To achieve this aim, we employed data from 537 ccRCC patients from TCGA dataset, using Spearman’s correlation. The list of top 25 genes correlate with MAP3K8 can be found in Supplementary Table 4, ranking by Spearman value. The most interesting identified target were displayed in Figure 6, including NFKBIZ (Spearman: 0.67, *P* = 4.44E-72), MIAT (Spearman: 0.67, *P* = 8.09E-71), PARP15 (Spearman: 0.66, *P* = 6.17E-68), CHFR (Spearman: 0.66, *P* = 4.47E-67), MKNK1 (Spearman: 0.65, *P* = 2.69E-66), and ERMN (Spearman: 0.65, *P* = 5.84E-65). cBioPortal was used to perform pathway analyses on coexpressed genes of MAP3K8. RTK-RAS, HIPPO, WNT, and Notch pathways are the most relevant pathways with scores of 35, 26, 25, and 19, respectively (Figure 7, Supplementary Figure 3, and Supplementary Table 5).

### Correlation of MAP3K8 With Tumor Immune Infiltrates (TILs)

**FIGURE 8:**
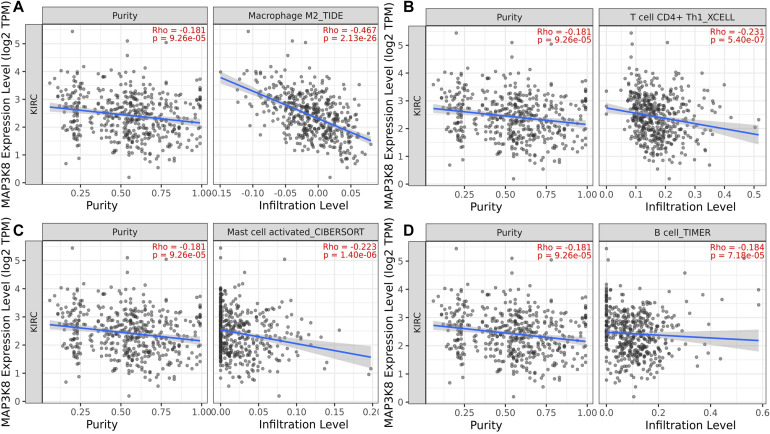
Negative correlation of MAP3K8 with tumor infiltration level of Macrophage M2 **(A)**, T cell CD4^+^ Th1 **(B)**, Mast cell activated **(C)**, and B cell **(D)** in ccRCC.

To determine whether the association between MAP3K8 expression and the presence of tumoral infiltrating immune cell populations exist in ccRCC samples, multiple immune deconvolution methods were applied. We found that expression level of MAP3K8 negatively correlated with the presence of macrophage M2 (TIDE: Rho = −0.47, *P* = 2.13E-26), T cell CD4^+^ Th1 (XCELL: Rho = −0.23, *P* = 5.40E-7), activated mast cell (CIBERSORT: Rho = −0.22, *P* = 1.40E-6), B cell (TIMER: Rho = −0.18, *P* = 7.18E-5), non-regulatory T cell CD4^+^ (QUANTISEQ: Rho = −0.15, *P* = 1.19E-3; XCELL: Rho = −0.10, *P* = 2.54E-2), plasma B cell (CIBERSORT: Rho = −0.15, *P* = 1.33E-3), NK cell (EPIC: Rho = −0.15, *P* = 1.40E-3), and activated mast cell (CIBERSORT-ABS: Rho = −0.12, *P* = 7.39E-3) (Figure 8 and Supplementary Table 6). In addition, expression level of MAP3K8 positively correlated with the presence of neutrophil (TIMER: Rho = 0.54, *P* = 6.61E-36), macrophage/monocyte (MCPCOUNTER: Rho = 0.45, *P* = 7.07E-24), monocyte (MCPCOUNTER: Rho = 0.45, *P* = 7.07E-24; XCELL: Rho = 0.34, *P* = 6.91E-14), macrophage M2 (CIBERSORT-ABS: Rho = 0.44, *P* = 4.77E-23), B cell (QUANTISEQ: Rho = 0.37, *P* = 9.23E-17), macrophage M1 (QUANTISEQ: Rho = 0.36, *P* = 5.80E-16), macrophage M2 (QUANTISEQ: Rho = 0.35, *P* = 5.30E-15), T cell regulatory (Tregs) (QUANTISEQ: Rho = 0.33, *P* = 1.59E-13) (Supplementary Table 7). In this study, these varied results indicated that associations between MAP3K8 expression and the presence of TILs in ccRCC samples may depend on methods used.

### PPI Networks and Functional Enrichment Analyses of MAP3K8

**FIGURE 9:**
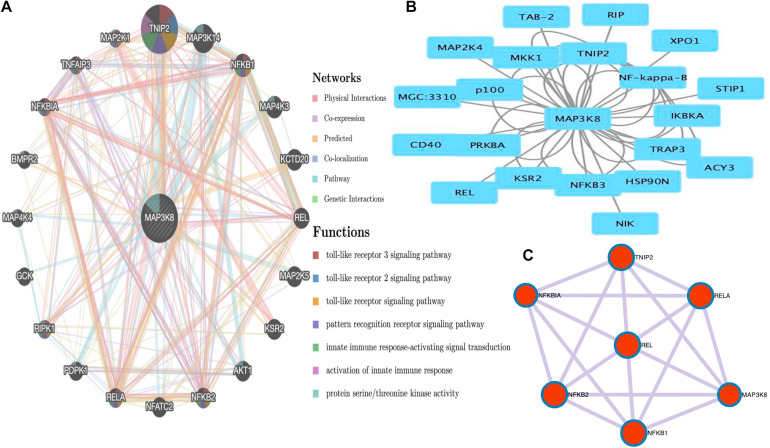
Gene Network Analysis. MAP3K8 gene with its neighboring genes involved in physical interactions, coexpression, predicted, co-localization, pathways, genetic interaction, and shared protein domains **(A)**. Protein-protein interaction (PPI) network of MAP3K8-correlated genes based on Cytoscape **(B)**. The core modules of genes and enriched terms associated with MAP3K8 through MCODE **(C)**.

**FIGURE 10:**
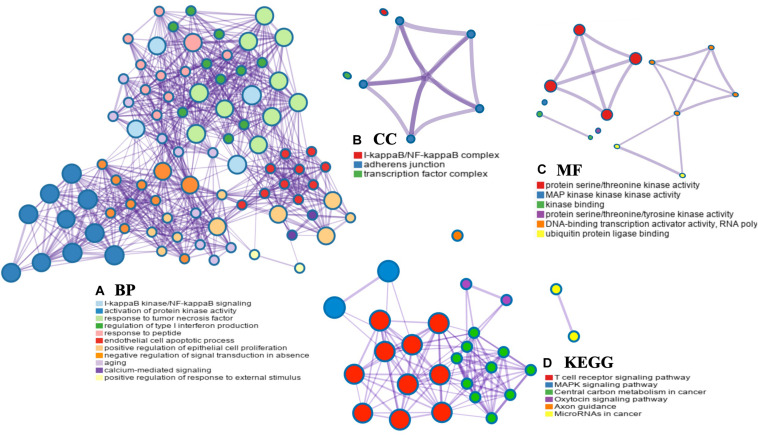
Network enrichment analysis for GO: biological process (BP) **(A)**, GO: cellular component (CC) **(B)**, GO: molecular function (MF) **(C)**, and Kyoto Encyclopedia of Genes and Genomes pathways (KEGG) **(D)** across MAP3K8 and other 21 most interactive genes using the Metascape server.

The PPI networks from the GeneMANIA website revealed a list of correlated genes for MAP3K8. The gene sets enriched for MAP3K8 were responsible for toll-like receptor signaling pathways, pattern recognition receptor signaling pathway, innate immune response-activating signal transduction, activation of innate immune response, and protein serine/threonine kinase activity (Figure 9A and Supplementary Table 8). In addition, the Cytoscape software was used to visualize the network of MAP3K8 by searching the BioGrid database. Each node, linked by edges, stood for an enriched term colored by the cluster-ID (Figure 9B). Meanwhile, a Cytoscape plugin-in in Metascape was used to construct network of core modules of genes, including REL, RELA, TNIP2, NFKB1, NFKB2, and NFKBIA (Figure 9C). GO and KEGG signal pathway analyses were conducted to identify the biological function of interactive genes of MAP3K8 derived from the Metascape website. In the diagram, different color represents the different biological process and the degree of color means the counts of enriched genes, in which the darker the color, the more genes were enriched in corresponding process. GO analysis found that the gene clusters had significant regulation of I-kappaB kinase/NF-kappaB signaling, activation of protein kinase activity, and response to tumor necrosis factor (Figures 10A–C and Supplementary Figures 4A–C). In KEGG enrichment analyses, T cell receptor signaling pathway, MAPK signaling pathway, and other biological pathways were identified for correlated genes of MAP3K8 (Figure 10D and Supplementary Figure 4D), which may suggest the underlying mechanism in the ccRCC pathogenesis.

## Discussion

Currently, efforts toward better understanding and characterizing molecular features underlying ccRCC tumorigenesis have been leveraged into new therapies targeting VEGF and immune checkpoint. Although a few patients may experience disease remission from tyrosine kinase inhibitor (TKI) and anti-PD-1, limited efficiency and resistance of drugs and disease progression are notable obstacles that remain to be solved (Escudier et al., 2019; Ljungberg et al., 2019). Previous studies have revealed that genetic alterations of MAP3K8 were involved in tumorigenic progression in colorectal cancer (Tunca et al., 2013), melanomas (Lehmann et al., 2019; Newman et al., 2019), and lung cancer (Boldrini et al., 2017). However, only little is known about the mechanisms underlying these phenomena of MAP3K8, especially in ccRCC.

This study described an association of MAP3K8 with ccRCC using computed methods. We found that MAP3K8 was overexpressed in ccRCC samples compared with normal kidney tissues and was a significant prognostic indicator for poor OS in ccRCC patients. In this study, we verified that, although mutational activation of MAP3K8 is a rare event, aberrant methylation may be a common alteration in ccRCC. Moreover, we found that MAP3K8 expression was associated with levels of some populations of immune infiltrates, along with enriched I-kappaB kinase (IKK)/NFKB and T cell receptor signaling pathways driven by MAP3K8 being the most significant biological process, suggesting a relevance for MAP3K8-related TILs. Therefore, these results will aid the understanding of tumor formation and progression of ccRCC and provide novel targets for cancer therapy.

Although MAP3K8 was originally recognized as an oncogene since its discovery in 1991 (Miyoshi et al., 1991), genetic sequence analyses identified rare MAP3K8 mutations which is much less than altered expression and abnormal activation in human cancers. Previous studies have found that overexpression and increased activation of MAP3K8 are main events associated with increased tumorigenesis including initiation, promotion, and progression as well as poor prognosis (Miyoshi et al., 1991; Sourvinos et al., 1999; Clark et al., 2004; Njunge et al., 2020). Because MAP3K8 in ccRCC has been less investigated, it is impossible to completely describe the functions. An important aspect of this study is the determination of clinical significance of MAP3K8 in ccRCC. By taking advantage of computed methods to overcome the challenges of fewness of clinical data, our results suggested that high level of MAP3K8 expression could predict poor OS in ccRCC, with damaged PFI and DSS, which were not displayed in this study. However, few published studies have verified how MAP3K8 played an oncogenic role in ccRCC.

Notably, more results with more comprehensive methods have been previously suggested in melanomas which is of great relevance to therapeutic targeting of MAPK cascades (Lehmann et al., 2019; Newman et al., 2019). Lehmann et al. (2019) identified recurrent MAP3K8 rearrangement in more than 15% of melanomas without known driver mutations using fluorescence *in situ* hybridization, only occurring in 1.7% of TCGA melanomas. However, MAP3K8 rearrangement is rarely reported in ccRCC. In melanomas, MAP3K8 overexpression could cause concomitant resistance to BRAF inhibitors through a MEK-dependent mechanism that does not require BRAF activation upstream, thus increasing sensitivity to MEK or ERK inhibitors (Johannessen et al., 2010; Lehmann et al., 2019; Newman et al., 2019). Therefore, MAP3K8 rearrangement could be a biomarker to identify melanoma patients that may benefit from MEK or ERK inhibitors. Given the fact that most genetic alterations of MAP3K8 in ccRCC is of no significance, further investigations will be necessary to incorporate MAP3K8 into assay design and focus on genetic fusion, rearrangement, amplification, or other genetic alterations conferring MAPK pathway dependency.

During cancer progression, tumor cells can alter intratumoral heterogeneity to orchestrate a generally supportive immunosuppressive tumor microenvironment (TME) comprised of populations of immune cells (Grivennikov et al., 2010), which have been reported as potential therapy targets for ccRCC in published studies (Motzer et al., 2015, 2018). Indeed, previous studies have confirmed that immunotherapy using immune checkpoint blockade, such as anti-PD-1 (Motzer et al., 2015, 2018) and anti-CTLA-4 (Motzer et al., 2018), to induce amplified endogenous anti-tumor T cell responses, has changed the clinical outcomes of ccRCC patients. Given the complexity of TME and multiple immune evasion mechanisms of solid tumors, intrinsic gene regulatory networks related to tumor-specific T cells should be deeply studied. MAP3K8 has been identified as an indispensable modulator of immune responses that conveys inflammatory signals, modulating functions of inflammatory cells (Yan et al., 2019). Specifically, MAP3K8 are found to regulate MEK/ERK/JNK, TLR, and IKK/NFKB cascades, which were particularly important for inflammatory responses (Lee et al., 2015a; Braicu et al., 2019; Yan et al., 2019). Yet, inflammatory responses play decisive roles at different stages of tumor development, and also affect immune surveillance and responses to anti-cancer therapy. Hence, principal mechanisms that mediate tumor-infiltrating immune cells need to be uncovered.

Conceivably, detecting and eliminating neoantigens released by cancer cells is highly dependent on an intricate innate immune system composed of sensors, signal-processing and signal-transducing elements, and myriad effector molecules (Grivennikov et al., 2010). Crucial to this process is the detection of pathogen-associated molecules by sensors, such as Toll-like receptor (TLR) (Barton and Medzhitov, 2003). The KEGG enrichment analyses in this study found that gene sets correlated with MAP3K8 were involved in the TLR signaling pathway. Previous studies confirmed that all TLRs activate a universal signaling pathway that culminates in the activation of ERK, JNK, and NFKB, which was again proved by Barton and Medzhitov (2003) and Banerjee et al. (2006) that MAP3K8/MEK/ERK pathway is required for ERK activation stimulated with TLRs. Regardless of how MAP3K8 affect TILs in ccRCC, the activation of TLRs could be expected to change antitumor immune responses of TILs. Activating TLRs through ligands not only reversed the suppressive function of CD4^+^ Treg cells but also blocked CD8^+^ Treg suppressive function, implying that manipulation of TILs by TLR signaling pathway could improve the efficacy of antitumor immune responses (Kiniwa et al., 2007). Moreover, studies have found that anti-tumor cytotoxicity of T lymphocytes could be promoted in cancer patients through activation of TLR-mediated MAPK and NFKB signaling pathways (Chen Y. et al., 2017), suggesting whether TLR pathway regulates functions of TILs via MAP3K8 cascades need to be further studied.

In this study, we have noted that MAP3K8 positively correlated with NFKBIZ, which is involved in regulation of NFKB transcription factor complexes and inhibits NFKB activity without affecting its nuclear translocation upon stimulation (DiDonato et al., 2012; Gantke et al., 2012). According to previous studies, the entire pool of MAP3K8 is positively associated with NFKB in steady state condition, irrespective of cell type, because NFKB binds to and masks the degron sequence of MAP3K8 preventing its proteasomal degradation, thus failing to phosphorylate MEK and ERK, while MAP3K8 expression is down-regulated in the absence of NFKB (Gantke et al., 2011; DiDonato et al., 2012; Gantke et al., 2012). Therefore, the associations between MAP3K8 and NFKBIZ in ccRCC need further researches. According to gene enrichment analyses in this study, MAP3K8 functions are responsible for the IKK/NFKB pathways. In addition to the aforementioned roles of NFKB in regulating MAP3K8, in some cases IKK complex, a central activator of the NFKB family, is also attributed to regulate MAP3K8 (Gantke et al., 2011, 2012; Hoesel and Schmid, 2013). In stable cells, MAP3K8 is complexed with the NF-κB1 p105 (NFKB1), a NFKB inhibitory protein, which blocks MAP3K8 access to its substrate MEK and the ubiquitin-binding protein ABIN-2, both of which are required to maintain MAP3K8 protein stability. In stimulated cells, the IKK complex phosphorylates NFKB1, triggering releases MAP3K8 from NFKB1-mediated inhibition, facilitating activation of MEK/ERK pathway. However, few studies have reported associations of IKK/NFKB pathways with TILs in cancers, but associations between TILs and TLR/MAPK pathways lay the foundation for further researches.

According to previous studies and the results in this study, effects of MAP3K8 on cancer-related inflammation in ccRCC may be double-edged. On one hand, ccRCC patients with high-level expression of MAP3K8 negatively correlated with the presence of T cell CD4^+^ Th1, non-regulatory T cell CD4^+^, and NK cell, suggesting a potential protumorigenic role of MAP3K8 because CD4^+^ TILs and NK cells were reported to associate with better OS in cancers (Hao et al., 2020). In addition, CD4^+^ Th1 secreting cytokines such as TNF may effectively inhibit angiogenesis as well as facilitate the activation and proliferation of CD8^+^ TILs, which could directly kill tumor cells (Ovarian Tumor Tissue Analysis [OTTA] Consortium, Goode et al., 2017; Hao et al., 2020). On the other hand, high expression level of MAP3K8 positively correlated with the presence of T cell regulatory. According to previous studies, Tregs exhibit tumor-promoting activity by limiting the development of autoimmunity and suppressing the function of Th1 cells, which may be inhibited to promote optimal antitumor responses (Kiniwa et al., 2007; Chen K. et al., 2017). Given the complexity of TME and multiple immune evasion mechanisms of solid tumors, intrinsic gene regulatory networks involved in activation, intratumoral trafficking, and persistence of tumor-specific T cells are complex. Thus, genetic mechanisms underlying regulating TILs in ccRCC remain elusive.

Finally, expression patterns and functions of MAP3K8 revealed in this study will promote the understanding of pathogenic mechanisms and exploration of therapeutic targets of ccRCC. The present study identified expression level and prognostic value of MAP3K8 in ccRCC using several databases. The results revealed that MAP3K8 overexpression was correlated with damaged survival in ccRCC, reemphasizing the potential for identifying predictive biomarkers and therapeutic targets focused on MAP3K8 and MAPK pathway. Our data also revealed several enriched functions that significantly associated with the MAP3K8. Moreover, this study reminds us of the potential roles of MAP3K8 in cancer-related immunity in ccRCC. Continuous studies will be required to power the discoveries of MAP3K8 in this study.

## Data Availability Statement

The original contributions presented in the study are included in the article/Supplementary Material, further inquiries can be directed to the corresponding author/s.

## Author Contributions

JH contributed to the study design and article writing. YC, HY, and LZ were responsible for figure editing. RA and YX were responsible for proofreading. All authors read and approved the final manuscript.

## Conflict of Interest

The authors declare that the research was conducted in the absence of any commercial or financial relationships that could be construed as a potential conflict of interest.

## Publisher’s Note

All claims expressed in this article are solely those of the authors and do not necessarily represent those of their affiliated organizations, or those of the publisher, the editors and the reviewers. Any product that may be evaluated in this article, or claim that may be made by its manufacturer, is not guaranteed or endorsed by the publisher.

## Footnotes

1
http://www.timer.comp-genomics.org

2
http://www.ualcan.path.uab.edu/index.html

3
https://www.oncomine.org/resource/login.html#

4
http://www.driverdb.tms.cmu.edu.tw

5
http://www.cbioportal.org

6
http://www.gepia.cancer-pku.cn/index.html

7
http://www.genemania.org

8
https://www.cytoscape.org

9
http://www.metascape.org/gp/index.html#/main/step1
