# Supplementary material for: Effect of MAP3K8 on Prognosis and Tumor-Related Inflammation in Renal Clear Cell Carcinoma
Source: Front Genet. 2021 Sep 10;12:674613. doi: 10.3389/fgene.2021.674613 (PMC8461076; doi:10.3389/fgene.2021.674613)
Supplement: Supplementary file 1 [file Data_Sheet_1.PDF]

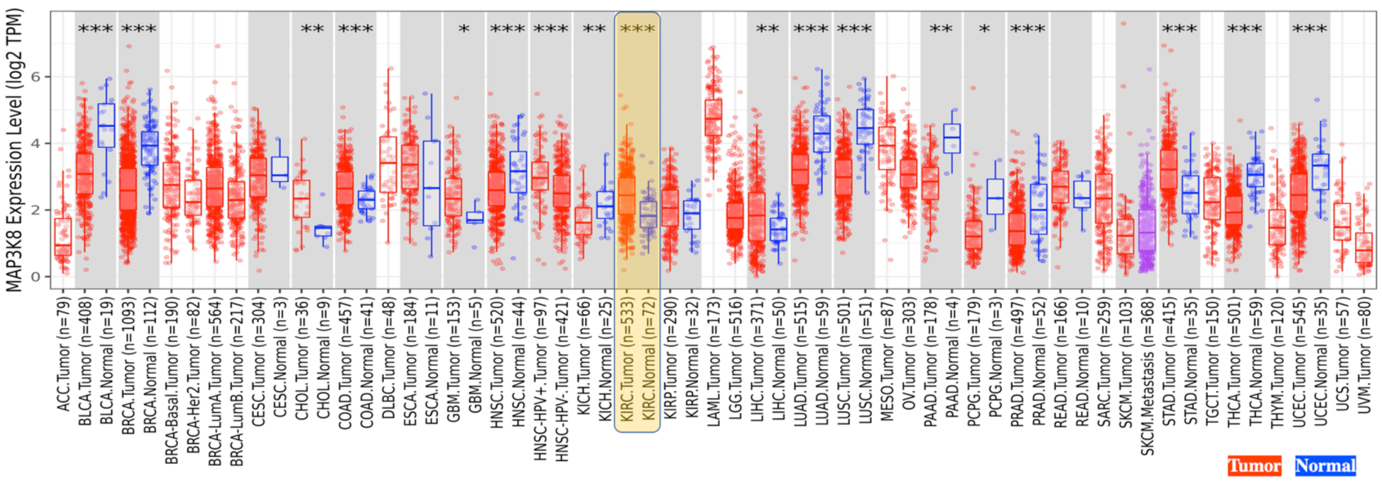

Supplementary Figure 1. Expression of MAP3K8 gene across different cancers where red and blue represent tumor and normal tissue, respectively. Statistical significance computed by differential analysis is annotated by the number of stars (*: P < 0.05; **: P < 0.01; ***P < 0.001). Box plot comparisons of MAP3K8 expression between tumor and normal tissue were from TIMER2.0.

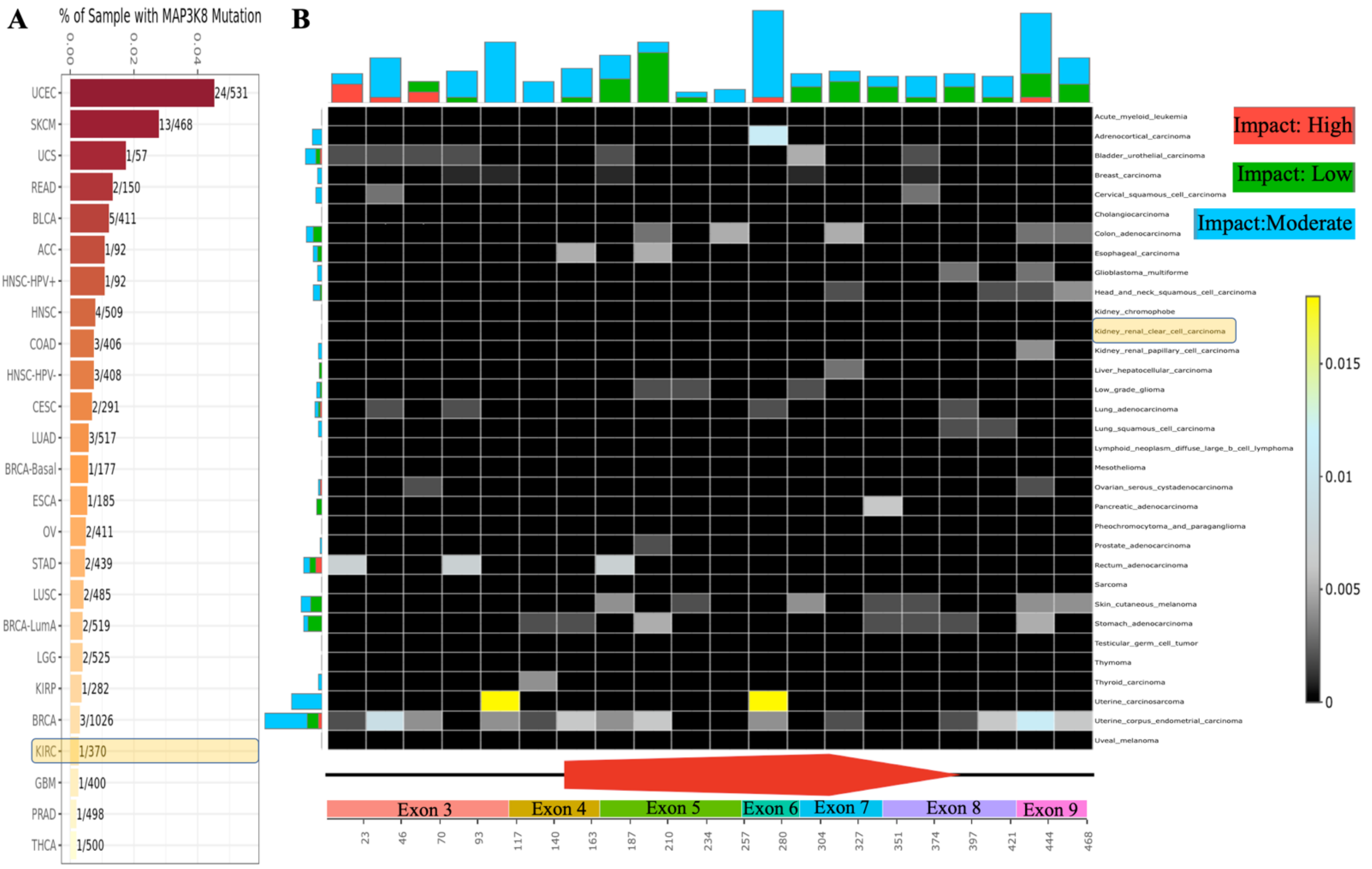

Supplementary Figure 2. Mutation status of MAP3K8 gene in different cancers. Percentage of sample with MAP3K8 mutation in different cancers from TIMER2.0 server **(A)**. Heatmap indicates the mutation rate of MAP3K8 gene at different protein positions in several cancer types from the DriverDBv3 server, and the mutation rate is the mutation count divided by sample count, which is indicated by color scale **(B)**.

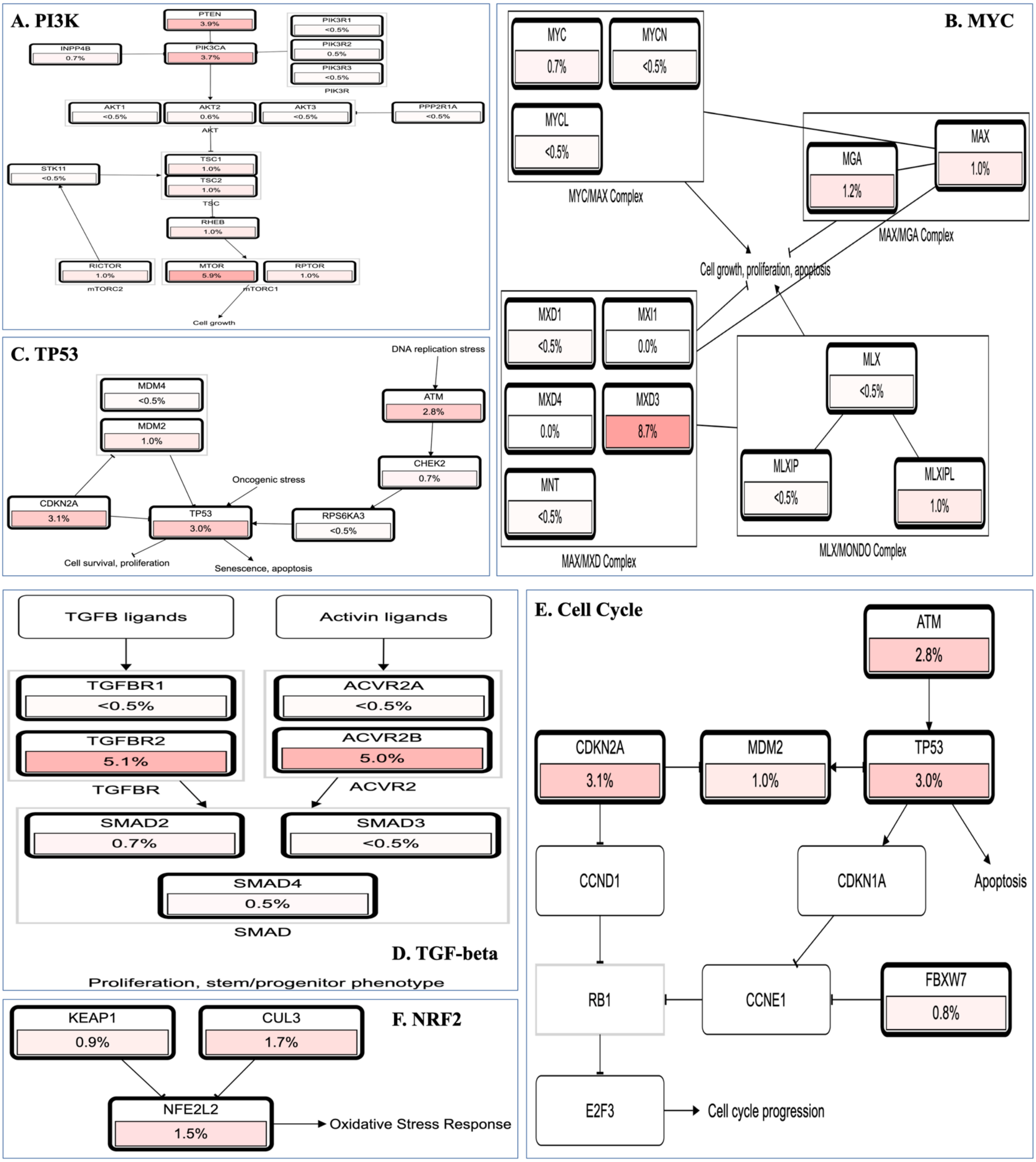

Supplementary Figure 3. Pathway analysis in kidney renal clear cell carcinoma (KIRC) using cBioPortal server. Impact of MAP3K8 and associated genes in regulating alteration frequency of PI3K signaling pathway **(A)**, MYC signaling pathway **(B)**, TP53 signaling pathway **(C),** TGF-beta signaling pathway **(D)**, cell cycle signaling pathway **(E)**, and NRF2 signaling pathway **(F)**.

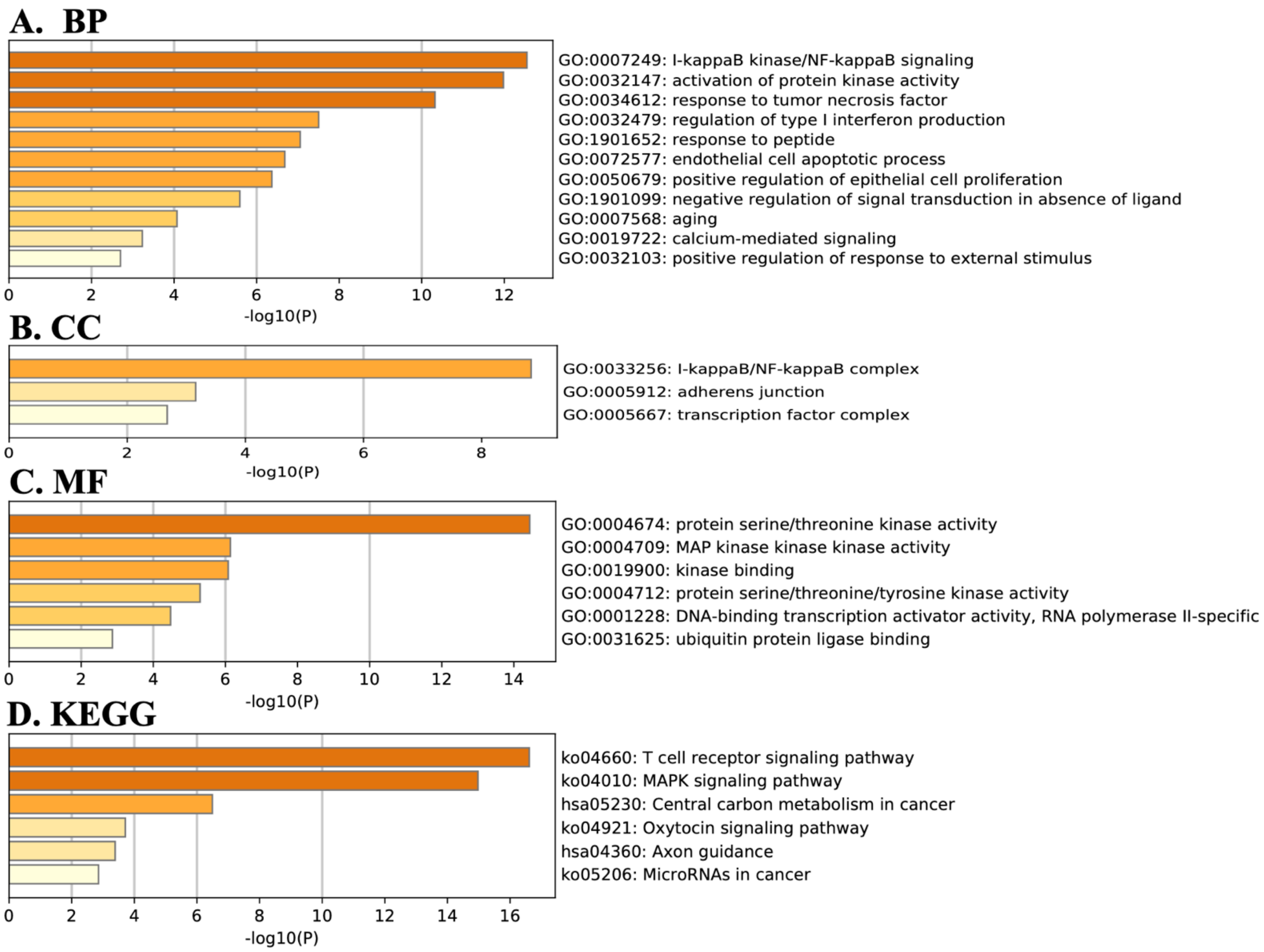

Supplementary Figure 4. Heatmaps of Gene Ontology (GO) analysis including biological process (BP) **(A)**, cellular component (CC) **(B)**, and molecular function (MF) **(C)**, and Kyoto Encyclopedia of Genes and Genomes pathways (KEGG) **(D)** across MAP3K8 and other 21 most interactive genes using the Metascape server. Orange is the enrichment terms and colored by *P* values.

Supplementary Table 1. Differential expression of MAP3K8 between tumor and adjacent normal tissues for all TCGA tumors using TIMER2.0 server. Bladder Urothelial Carcinoma (BLCA), Breast invasive carcinoma (BRCA), Cervical squamous cell carcinoma and endocervical adenocarcinoma (CESC), Cholangio carcinoma (CHOL), Colon adenocarcinoma (COAD), Esophageal carcinoma (ESCA), Glioblastoma multiforme (GBM), Head and Neck squamous cell carcinoma (HNSC), Kidney Chromophobe (KICH), Kidney renal clear cell carcinoma (KIRC), Kidney renal papillary cell carcinoma (KIRP), Liver hepatocellular carcinoma (LIHC), Lung adenocarcinoma (LUAD), Lung squamous cell carcinoma (LUSC), Pancreatic adenocarcinoma (PAAD), Pheochromocytoma and Paraganglioma (PCPG), Prostate adenocarcinoma (PRAD), Rectum adenocarcinoma (READ), Skin Cutaneous Melanoma (SKCM), Stomach adenocarcinoma (STAD), Thyroid carcinoma (THCA), Uterine Corpus Endometrial Carcinoma (UCEC)

**Tumor**	**Normal**	***P*-Value**
BLCA.Tumor (n=408)	BLCA.Normal (n=19)	3.46E-06
BRCA.Tumor (n=1093)	BRCA.Normal (n=112)	2.02E-33
CESC.Tumor (n=304)	CESC.Normal (n=3)	5.90E-01
CHOL.Tumor (n=36)	CHOL.Normal (n=9)	1.77E-03
COAD.Tumor (n=457)	COAD.Normal (n=41)	1.18E-04
ESCA.Tumor (n=184)	ESCA.Normal (n=11)	3.40E-01
GBM.Tumor (n=153)	GBM.Normal (n=5)	4.38E-02
HNSC.Tumor (n=520)	HNSC.Normal (n=44)	4.49E-04
HNSC-HPV+Tumor (n=97)	HNSC-HPV-Tumor (n=421)	7.58E-07
KICH.Tumor (n=66)	KICH.Normal (n=25)	4.38E-03
KIRC.Tumor (n=533)	KIRC.Normal (n=72)	2.61E-10
KIRP.Tumor (n=290)	KIRP.Normal (n=32)	7.10E-02
LIHC.Tumor (n=371)	LIHC.Normal (n=50)	4.57E-03
LUAD.Tumor (n=515)	LUAD.Normal (n=59)	1.12E-16
LUSC.Tumor (n=501)	LUSC.Normal (n=51)	1.98E-22
PAAD.Tumor (n=178)	PAAD.Normal (n=4)	8.67E-03
PCPG.Tumor (n=179)	PCPG.Normal (n=3)	4.79E-02
PRAD.Tumor (n=497)	PRAD.Normal (n=52)	4.68E-05
READ.Tumor (n=166)	READ.Normal (n=10)	1.85E-01
SKCM.Tumor (n=103)	SKCM.Metastasis (n=368)	9.40E-02
STAD.Tumor (n=415)	STAD.Normal (n=35)	1.59E-06
THCA.Tumor (n=501)	THCA.Normal (n=59)	6.74E-23
UCEC.Tumor (n=545)	UCEC.Normal (n=35)	4.90E-06

Supplementary Table 2. The MAP3K8 mRNA expression for kidney renal clear cell carcinoma (KIRC) based on different clinicopathologic parameters using UALCAN server.

**Variables**	**Subgroup**	**No.**	**Comparisons**	**Statistical significance**
Sample types	Normal	72	Normal vs. primary tumor	6.88E-15
	Primary tumor	533		
Cancer stage	Normal	72		
	Stage 1	267	Normal vs. Stage 1	9.80E-12
	Stage 2	57	Normal vs. Stage 2	1.80E-04
	Stage 3	123	Normal vs. Stage 3	1.17E-08
	Stage 4	84	Normal vs. Stage 4	4.84E-06
Patient's gender	Normal	72		
	Male	345	Normal vs. Male	9.10E-14
	Female	188	Normal vs. Female	1.23E-09
Patient's age	Normal	72		
	21-40 years	26		
	41-60 years	238	Normal vs. Age (41-60)	8.49E-13
	61-80 years	246	Normal vs. Age (61-80)	5.34E-11
	81-100 years	23	Normal vs. Age (81-100)	7.74E-05
Patient's race	Normal	72		
	Caucasian	462	Normal vs. Caucasian	2.62E-14
	African-american	56	Normal vs. African-american	2.63E-04
	Asian	8	Normal vs. Asian	5.95E-04
KIRC subtypes	Normal	72	Normal vs. ccA	4.97E-08
	ccA	205	Normal vs. ccB	2.39E-12
	ccB	175	ccA vs. ccB	8.77E-04
Tumor grade	Normal	72	Normal vs. Grade 1	1.37E-02
	Grade 1	14	Normal vs. Grade 2	3.06E-10
	Grade 2	229	Normal vs. Grade 3	8.84E-11
	Grade 3	206	Normal vs. Grade 4	8.01E-07
	Grade 4	76	Grade 3 vs. Grade 4	2.42E-02
Nodal metastasis	Normal	72		
	N0	240	Normal vs. N0	3.99E-11
	N1	16		

Supplementary Table 3. The MAP3K8 promoter methylation for kidney renal clear cell carcinoma (KIRC) based on different clinicopathologic parameters using UALCAN server.

Variables	Subgroup	No.	Comparisons	Statistical significance
Sample types	Normal	160	Normal vs. primary tumor	<1E-12
	Primary tumor	324		
Patient's race	Normal	160		
	Caucasian	271	Normal vs. Caucasian	1.11E-16
	African-american	49	Normal vs. African-american	1.71E-05
	Asian	1		
Patient's gender	Normal	160		
	Male	210	Normal vs. Male	4.91E-12
	Female	114	Normal vs. Female	5.26E-12
Patient's age	Normal	160		
	21-40 years	12	Normal vs. Age (21-40)	9.95E-05
	41-60 years	141	Normal vs. Age (41-60)	2.13E-10
	61-80 years	154	Normal vs. Age (61-80)	5.96E-12
	81-100 years	17	Normal vs. Age (81-100)	4.86E-02
Tumor grade	Normal	160	Normal vs. Grade 1	5.20E-03
	Grade 1	9	Normal vs. Grade 2	1.03E-11
	Grade 2	138	Normal vs. Grade 3	2.07E-07
	Grade 3	123	Normal vs. Grade 4	2.61E-07
	Grade 4	50	Grade 1 vs. Grade 2	1.28E-03
			Grade 1 vs. Grade 3	7.41E-04
Nodal metastasis	Normal	160		
	N0	135	Normal vs. N0	2.48E-12
	N1	8		

Supplementary Table 4. Coexpression and Correlation of the top 25 gene associated with MAP3K8 gene in kidney renal clear cell carcinoma (KIRC) using cBioPortal. The p-value is derived from two-sided t-test and the q-value derived from Benjamini-Hochberg FDR correction procedure.

**Correlated Gene**	**Cytoband**	**Spearman's Correlation**	**p-Value**	**q-Value**
NFKBIZ	3q12.3	0.674	4.44E-72	8.96E-68
MIAT	22q12.1	0.67	8.09E-71	8.16E-67
PARP15	3q21.1	0.659	6.17E-68	4.15E-64
CHFR	12q24.33	0.656	4.47E-67	2.25E-63
MKNK1	1p33	0.653	2.69E-66	1.09E-62
ERMN	2q24.1	0.648	5.84E-65	1.96E-61
STX16	20q13.32	0.638	2.99E-62	8.62E-59
WSB1	17q11.1	0.632	5.73E-61	1.44E-57
FNBP4	11p11.2	0.628	5.91E-60	1.32E-56
KLRA1P	12p13.2	0.626	1.47E-59	2.91E-56
PARVG	22q13.31	0.626	1.59E-59	2.91E-56
TRAF5	1q32.3	0.626	1.96E-59	3.30E-56
HSPA7	1q23.3	0.625	2.91E-59	4.52E-56
SNORA8	11q21	0.625	3.79E-59	5.47E-56
PLCB2	15q15.1	0.623	1.01E-58	1.36E-55
HSPA6	1q23.3	0.611	4.77E-56	6.01E-53
WNT10B	12q13.12	0.611	6.55E-56	7.78E-53
RBM33	7q36.3	0.608	3.26E-55	3.65E-52
VAMP1	12p13.31	0.605	1.24E-54	1.32E-51
ODF2L	1p22.3	0.604	1.78E-54	1.80E-51
SLC7A6	16q22.1	0.602	4.59E-54	4.27E-51
AGAP6	10q11.23	0.602	4.66E-54	4.27E-51
GCNA	Xq13.1	0.601	9.11E-54	7.99E-51
KIF21B	1q32.1	0.601	1.00E-53	8.44E-51
ARRDC5	19p13.3	0.597	9.09E-53	7.34E-50

Supplementary Table 5. Scores and matched genes of the pathways in kidney renal clear cell carcinoma (KIRC) using cBioPortal server.

**Pathway name**	**Score**	**Genes matched**
RTK-RAS	35	NRAS, ROS1, KIT, PTPN11, RET, CBL, FLT3, NF1, NTRK2, ERRFI1, ARAF, FGFR3, HRAS, MET, RAF1, RAC1, FGFR1, FGFR2, KRAS, BRAF, ERBB2, ERBB4, MAPK1, EGFR, RASA1, FGFR4, IGF1R, ERBB3, SOS1, RIT1, MAP2K2, ALK, NTRK1, PDGFRA, MAP2K1
HIPPO	26	DCHS1, DCHS2, FAT1, FAT2, FAT3, FAT4, TAOK1, TAOK3, TAOK2, SAV1, STK3, STK4, LATS1, LATS2, MOB1A, MOB1B, NF2, WWC1, YAP1, TAZ, CRB1, CRB2, PTPN14, CSNK1E, CSNK1D, TEAD2
WNT	25	TLE1, TLE4, SFRP1, APC, TCF7L1, WIF1, TLE2, DKK1, TCF7L2, AXIN1, SFRP2, LRP5, TCF7, TLE3, RNF43, CTNNB1, DKK2, DKK4, LRP6, GSK3B, SFRP5, AMER1, SFRP4, AXIN2, DKK3
NOTCH	19	NRARP, CUL1, CREBBP, NCOR2, SPEN, NOTCH3, CNTN6, KAT2B, KDM5A, DNER, NCOR1, FBXW7, NOTCH1, JAG2, PSEN2, EP300, NOTCH2, ARRDC1, NOTCH4
PI3K	17	AKT3, RPTOR, PIK3CA, PIK3R2, RHEB, AKT2, PPP2R1A, PIK3R3, PTEN, MTOR, PIK3R1, INPP4B, TSC1, TSC2, RICTOR, AKT1, STK11
MYC	13	MXD1, MXD4, MXI1, MGA, MYC, MYCL, MLXIPL, MAX, MXD3, MLXIP, MLX, MYCN, MNT
TP53	7	CDKN2A, MDM4, RPS6KA3, MDM2, TP53, ATM, CHEK2
TGF-Beta	7	SMAD2, ACVR2B, ACVR2A, SMAD3, TGFBR2, TGFBR1, SMAD4
Cell Cycle	5	FBXW7, CDKN2A, MDM2, TP53, ATM
NRF2	3	KEAP1, CUL3, NFE2L2

Supplementary Table 6. Negative correlation of MAP3K8 expression with 9 immune infiltrates in kidney renal clear cell carcinoma (KIRC) using TIMER, MCP-COUNTER, CIBERSORT, QUANTISEQ, and xCell algorithms. Purity Adjustment was used to adjust purity for the asscociation analysis.

**cancer**	**infiltrates**	**rho**	**p**	**adj.p**
KIRC (n=533)	Macrophage M2_TIDE	-0.47	2.13E-26	1.28E-24
KIRC (n=533)	T cell CD4+ Th1_XCELL	-0.23	5.40E-07	5.67E-06
KIRC (n=533)	Mast cell activated_CIBERSORT	-0.22	1.40E-06	3.06E-05
KIRC (n=533)	B cell_TIMER	-0.18	7.18E-05	5.67E-04
KIRC (n=533)	T cell CD4+ (non-regulatory)_QUANTISEQ	-0.15	1.19E-03	5.63E-03
KIRC (n=533)	B cell plasma_CIBERSORT	-0.15	1.33E-03	6.71E-03
KIRC (n=533)	NK cell_EPIC	-0.15	1.40E-03	7.70E-03
KIRC (n=533)	Mast cell activated_CIBERSORT-ABS	-0.12	7.39E-03	3.21E-02
KIRC (n=533)	T cell CD4+ (non-regulatory)_XCELL	-0.10	2.54E-02	7.13E-02

Supplementary Table 7. Positive correlation of MAP3K8 expression with top 9 immune infiltrates in kidney renal clear cell carcinoma (KIRC) using TIMER, MCP-COUNTER, CIBERSORT, QUANTISEQ, and xCell algorithms. Purity Adjustment was used to adjust purity for the asscociation analysis.

**cancer**	**infiltrates**	**rho**	**p**	**adj.p**
KIRC (n=533)	Neutrophil_TIMER	0.54	6.61E-36	3.95E-34
KIRC (n=533)	Macrophage/Monocyte_MCPCOUNTER	0.45	7.07E-24	2.80E-22
KIRC (n=533)	Monocyte_MCPCOUNTER	0.45	7.07E-24	2.80E-22
KIRC (n=533)	Macrophage M2_CIBERSORT-ABS	0.44	4.77E-23	1.51E-21
KIRC (n=533)	B cell_QUANTISEQ	0.37	9.23E-17	2.77E-14
KIRC (n=533)	Macrophage M1_QUANTISEQ	0.36	5.80E-16	9.15E-15
KIRC (n=533)	Macrophage M2_QUANTISEQ	0.35	5.30E-15	7.95E-14
KIRC (n=533)	Monocyte_XCELL	0.34	6.91E-14	6.58E-13
KIRC (n=533)	T cell regulatory (Tregs)_QUANTISEQ	0.33	1.59E-13	4.25E-12

Supplementary Table 8. Top 10 functions of 21 correlated genes of MAP3K8 using GeneMANIA server.

**Function**	**FDR**	**Genes in network**	**Genes in genome**
toll-like receptor 3 signaling pathway	2.81E-10	8	83
toll-like receptor 2 signaling pathway	7.99E-09	7	77
toll-like receptor signaling pathway	7.99E-09	8	137
pattern recognition receptor signaling pathway	1.21E-08	8	158
innate immune response-activating signal transduction	1.21E-08	8	160
activation of innate immune response	1.50E-08	8	168
protein serine/threonine kinase activity	2.13E-08	9	290
regulation of I-kappaB kinase/NF-kappaB signaling	2.45E-08	8	185
toll-like receptor 4 signaling pathway	2.55E-08	7	107
positive regulation of innate immune response	2.55E-08	8	191
